# The role of microbiota in autism spectrum disorder: A bibliometric analysis based on original articles

**DOI:** 10.3389/fpsyt.2022.976827

**Published:** 2022-09-12

**Authors:** Xiaoling Lin, Runjin Zhou, Dandan Liang, Lingling Xia, Liying Zeng, Xiaogang Chen

**Affiliations:** ^1^The First Clinical Medical College of Guangzhou University of Chinese Medicine, Guangzhou, China; ^2^Medical College of Acupuncture-Moxibustion and Rehabilitation, Guangzhou University of Chinese Medicine, Guangzhou, China; ^3^The First Affiliated Hospital of Guangzhou University of Chinese Medicine, Guangzhou, China

**Keywords:** microbiota, autism spectrum disorder, CiteSpace, bibliometric analysis, data visualization

## Abstract

**Background:**

Gastrointestinal (GI) symptoms can be observed in autism spectrum disorder (ASD) children. It is suggested that the gut microbiota and its metabolites are associated, not only with GI symptoms, but also with behaviors of ASD. The aim of this study was to explore the development context, research hotspots and frontiers of gut microbiota and ASD from January 1, 1980 to April 1, 2022 by bibliometric analysis.

**Materials and methods:**

Publications of ASD and gut microbiota research from 1 January 1980 to 1 April 2022 were retrieved from the Web of Science Core Collection (WoSCC). Publications and citations trends were analyzed by Excel 2010. CiteSpace was used to analyze countries/regions, authors, institutes, references, and keywords and to visualize the knowledge map.

**Results:**

A total of 1027 studies were retrieved, and 266 original articles were included after screening. The most published countries and institutes were the United States and King Saud University. Afaf El-Aansary published the most articles, while Finegold SM had the highest co-citations. Hotspots and emerging trends in this area may be indicated by co-cited references and keywords and their clusters, including “gut-brain axis,” “behavior,” “chain fatty acid,” “brain,” “feces,” “propionic acid,” “clostridium perfringens,” and “species clostridium innocuum.”

**Conclusion:**

The United States dominants the research in this field, which focuses on the alterations of gut microbiota composition and its metabolites, among which the roles of the genus *Clostridium* and metabolites of short-chain fatty acids, especially propionic acid, are priorities. Fecal microbiota transplantation (FMT) is a promising complementary therapy. In general, research in this area is sparse, but it still has great research prospects.

## Introduction

Autism spectrum disorder (ASD) is a neurodevelopmental disorder characterized by deficits in social communication, social interaction and restricted, repetitive patterns of behavior, interest or activities. These impairments occur in the early stage of life. The United States Centers for Disease Control and Prevention (CDC) released the latest autism prevalence report in 2021, analyzing the data of the Autism and Developmental Disabilities Monitoring Network (ADDM) in 2018. It is estimated that one in 44 children aged 8 in the United States has autism (1). Although many studies have focused on the pathogenesis of ASD, the exact mechanism remains unclear. Treatments for ASD are still limited.

Gastrointestinal (GI) distress is one of the most common comorbidities in ASD. Feeding or eating problems existed in 7 of the 11 children with autism described in the first description of autism by Kanner (2), which was seen as evidence of an early link between autism and the gastrointestinal tract. The incidence of GI symptoms in ASD is four times higher than that in the control group (3), and it is related to the severity of ASD core symptoms (4). The cause of GI symptoms in ASD remains unclear, but gut microbiota may play an important role because the composition and metabolites of gut microbiota change in both autistic patients and animals (5–9).

The gut microbiota is a community of bacteria colonized in the human intestines and interdependent with the human body, including more than 40 genera and 400 to 500 species. Research over the past years indicates that the gut microbiota has an effect on neurodevelopment. It has been reported that the maternal microbiome may modulate fetal neurodevelopment in mice through signaling by microbe-related metabolites in developing brain neurons (10). Major processes of neurodevelopment are consistent with alterations in the gut microbiota of the mother and newborn (11). Altered expression of neuroreceptors (12, 13) and NMDA receptor subunits (14), along with impaired blood–brain barrier function (15) and increased prefrontal cortex myelination (16), were observed in the hippocampus of germ-free (GF) mice. Furthermore, the gut microbiota can modulate animal behaviors. Sharon et al. (17) transplanted the gut microbiota from ASD patients into GF mice, and the mice developed autistic symptoms.

Based on the brain-gut axis theory and genome sequencing technology, the association between ASD and gut microbiota has been explored. However, research on ASD and gut microbiota is scattered and inconclusive. Therefore, in this study, CiteSpace, an information visualization and bibliometric analysis software, was used to review studies in this domain from January 1, 1980 to April 1, 2022 to evaluate the development context, research trends and future frontiers.

## Materials and methods

### Data source and retrieval strategy

Literature retrieval was completed online through the Science Citation Index-Expanded (SCI-E) of the Web of Science Core Collection (WoSCC) on the 1st April 2022. The retrieval strategy was as follows: TS = ((microbiome* OR microbiota* OR flora* OR microbe* OR mycobiome*) AND (Autism Spectrum Disorder* OR Autistic Spectrum Disorder* OR Autistic Disorder* OR Autism)) AND Language = English, Document Types = Article, Time range = January 1, 1980–April 1, 2022.

### Data collection

Initial data were downloaded from WoSCC in a plain text format, including full records and references cited. Then, the data were verified by two members (Runjin Zhou and Dandan Liang), and only original articles were included in this study. Articles that met the inclusion criteria were imported into CiteSpace V.5.5. R3 (Drexel University, Philadelphia, PA, United States). The information generated by CiteSpace was imported into Excel 2010 (Redmond, WA, United States).

### Data analysis

The characteristics of the dataset were analyzed by WoSCC, including research fields, journal sources, number of impact factors and citations and annual publications. Publications and citations trends were analyzed by Excel 2010.

CiteSpace was used to analyze and visualize trends and patterns in scientific papers through scientometrics, data and information visualization. It conducts the analysis of countries/regions, co-citations, keywords, authors and institutes and visualizes the knowledge maps. In the generated knowledge mapping, the larger the node is, the more important the content. The line between nodes indicates a connection. The thicker the line is, the stronger the connection. The interpretation of the generated knowledge mapping mainly focuses on the high-frequency nodes, clusters, nodes of high centrality and the basic legend of the mapping.

## Results

**FIGURE 1:**
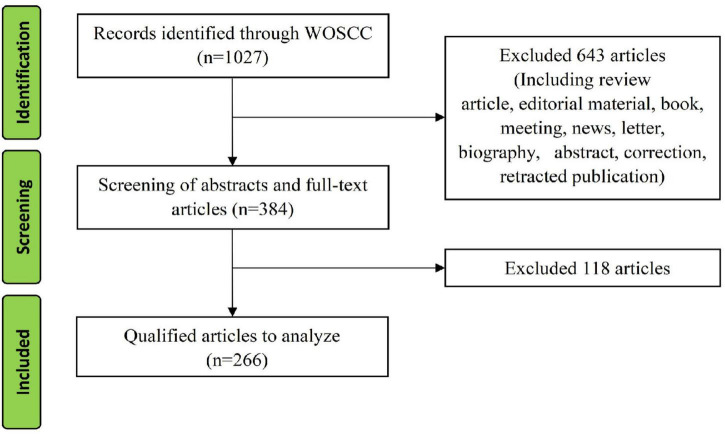
Study flow diagram.

A total of 1027 studies were retrieved from January 1, 1980 to April 1, 2022, and 266 original articles were included after screening. The detailed screening process is shown in Figure 1.

### Analysis of publications and citations

**FIGURE 2:**
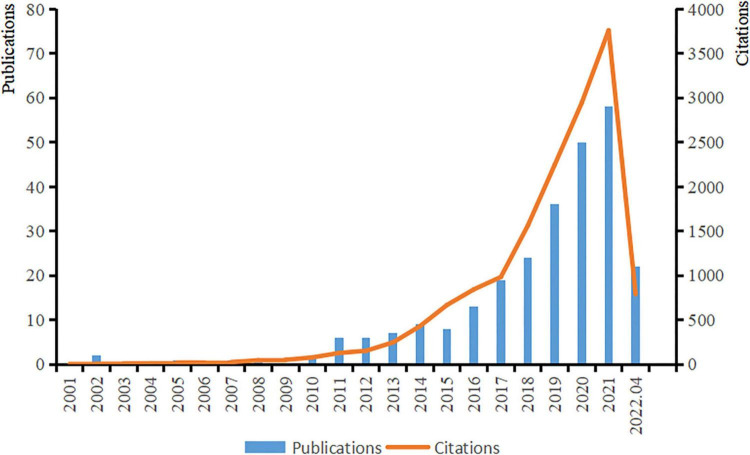
Trends in publications and citations from January 1980 to April 2022.

The first article on ASD and microbiota appeared in 2000, and the number of studies in this field increased rapidly until 2010, with an increasing trend year by year. With the increase in attention and research on autism, the number of citations has tended to grow rapidly and is still growing (Figure 2).

### Distribution of countries/regions and institutes

**FIGURE 3:**
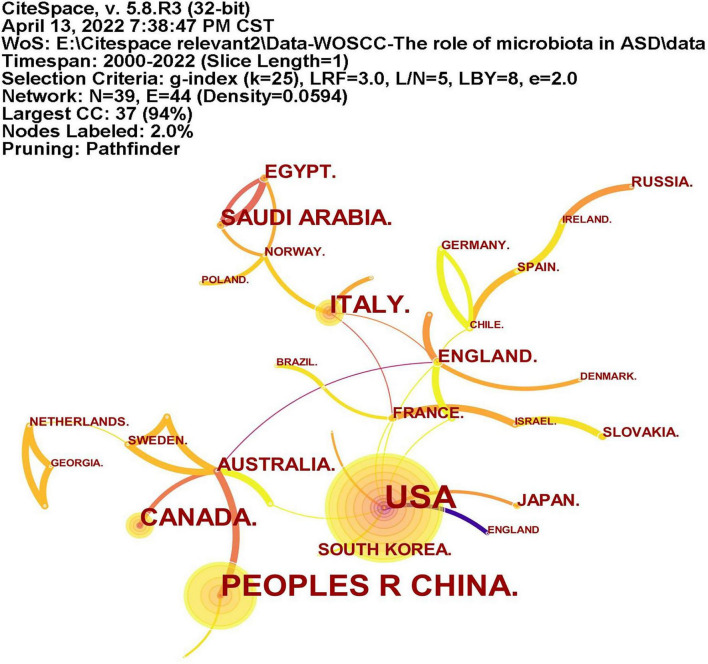
The network of countries/regions publications and collaborations. The circles represent the number of publications and lines represent collaborations. Larger circles represent more articles published and thicker lines indicate closer collaborations.

A total of 39 countries/regions have published articles on the association between ASD and microbiota (Figure 3). The circles in figure represent the number of publications and lines represent collaborations. Larger circles indicate more articles published and thicker lines indicate closer collaborations. Table 1 lists the top 10 countries in terms of the number of publications, among which the United States has the most research, followed by China, Italy, Canada, and Saudi Arabia. As shown in Figure 3, the United States, Britain, France and Australia cooperated more with other countries.

**TABLE 1:** The top 10 countries and institutions on publications in ASD and microbiota research.

Rank	Country	Count	Institutes	Count
1	United States	85	King Saud University	18
2	PR China.	62	Arizona State University	13
3	Italy.	26	National Research Center	10
4	Canada.	24	Fudan University	7
5	Saudi Arabia.	18	Massachusetts General Hospital	6
6	Australia.	12	Chinese Academy of Sciences	6
7	Egypt.	12	Princess Nourah Bint Abdulrahman University	6
8	England.	10	University of California Davis	6
9	Japan.	8	Baylor College Med	6
10	South Korea.	8	Chongqing Medical University	5

**FIGURE 4:**
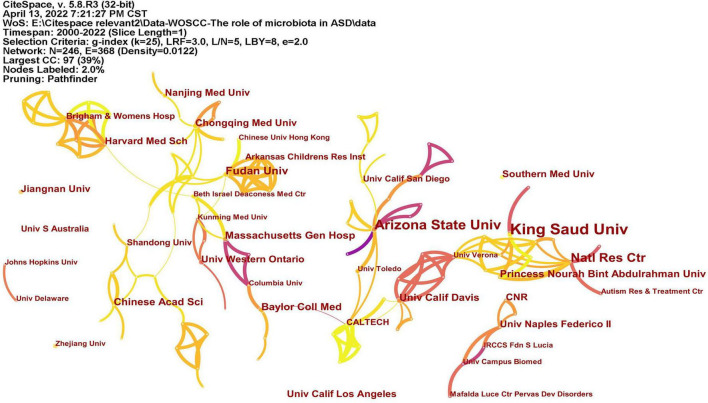
The network of institutes with articles published. The lines represent the collaborations.

A total of 246 institutions participated in research in this field (Figure 4). Table 1 lists the top 10 institutions with the most publications, among which King Saud University had published most articles. In terms of national distribution, four of them are from the United Sates, three are from China, two are from Saudi Arabia and the remaining one is from Egypt.

### Distribution of authors

**FIGURE 5:**
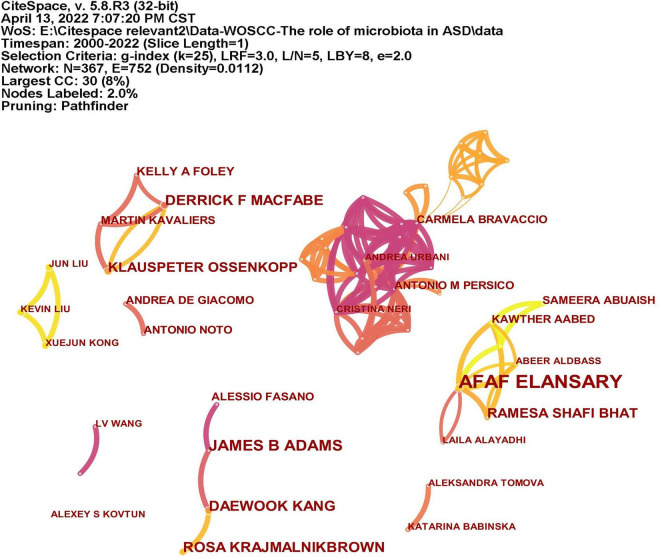
The network of authors publications and collaborations. The size of the letters represents the number of publications and the lines represent collaborations. The larger the letter is, the greater the number of publications. Thicker lines indicate closer collaborations.

A total of 366 researchers made contributions to the research on ASD and microbiota (Figure 5). The size of the letters in figure represents the number of publications and the lines represent collaborations. The larger the letter is, the greater the number of publications. Thicker lines indicate closer collaborations. The most productive author was Afaf El-Ansary (*n* = 16) from Saudi Arabia, followed by James B. Adams (*n* = 8), Dae-Wook Kang (*n* = 7), and Rosa Krajmalnik-Brown (*n* = 7) all from the United States, and Derrick F. Macfabe (*n* = 7) from Canada. Table 2 lists the top 10 authors in the number of publications.

**TABLE 2:** The top 10 authors and co-cited authors.

Rank	Author	Count	Co-cited author	Count
1	Afaf Elansary	16	Finegold SM	139
2	James B. Adams	8	Kang DW	113
3	Dae-Wook Kang	7	Adams JB	105
4	Derrick F. Macfabe	7	Hsiao EY	100
5	Rosa Krajmalnikbrown	7	Wang L	92
6	Ramesa Shafi Bhat	6	de Angelis M	90
7	Klauspeter Ossenkopp	6	Parracho HMRTt	87
8	Kelly A. Foley	5	Tomova A	66
9	Antonio M. Persico	4	Williams BL	65
10	Alessio Fasano	4	Strati F	60

**FIGURE 6:**
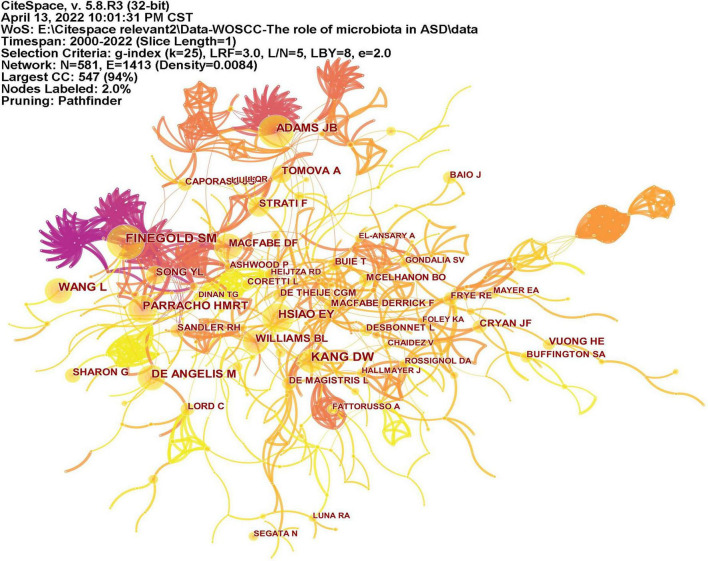
The network of co-cited authors of publications. Lines indicate that two authors are jointly cited in an article. Line thickness is proportional to the number of co-cited articles.

CiteSpace analyzed the author citation information to explore the academic relationship between authors and visualized it in a network (Figure 6). Line thickness is proportional to the number of co-cited articles. Table 2 lists the top 10 co-cited authors, among which the most co-cited author is Finegold SM from the United States (*n* = 139), followed by Kang DW (*n* = 113), Adams JB (*n* = 105), and Hsiao EY (*n* = 100) all from the United States, and Wang L (*n* = 92) from Australia.

### Analysis of keywords

**FIGURE 7:**
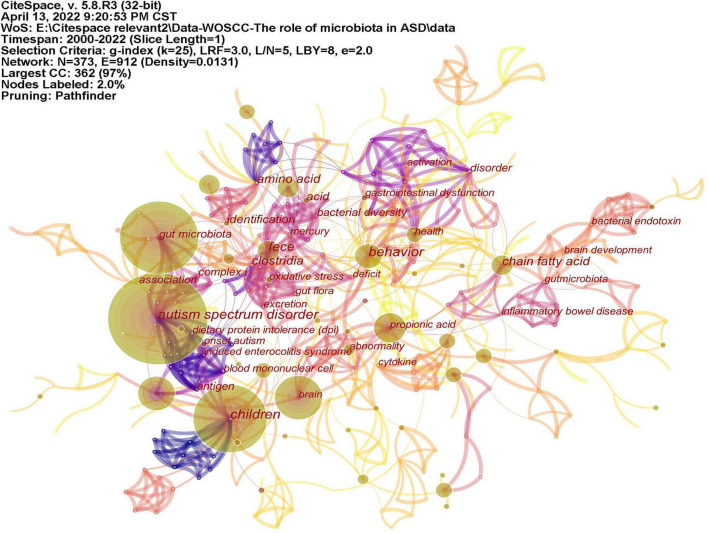
The network of keywords on microbiota and ASD. The nodes represent keywords and the size of a node is proportional to the frequency of keyword occurrence.

**FIGURE 8:**
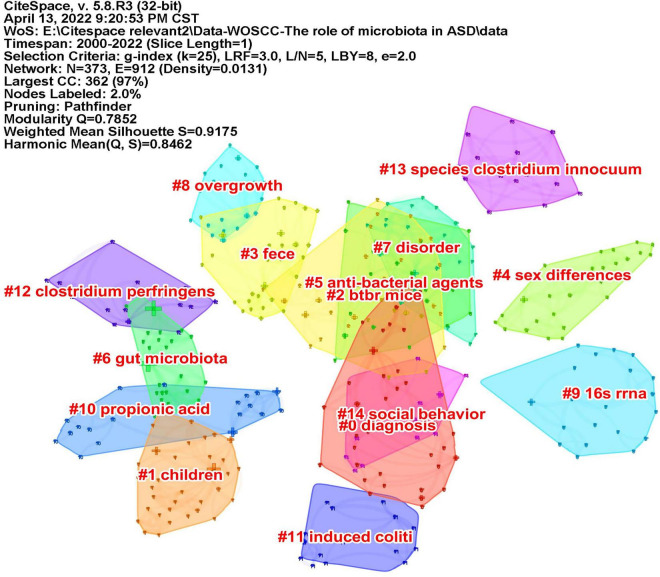
The map of keyword clusters. The clusters are arranged in the descending order of their size.

**FIGURE 9:**
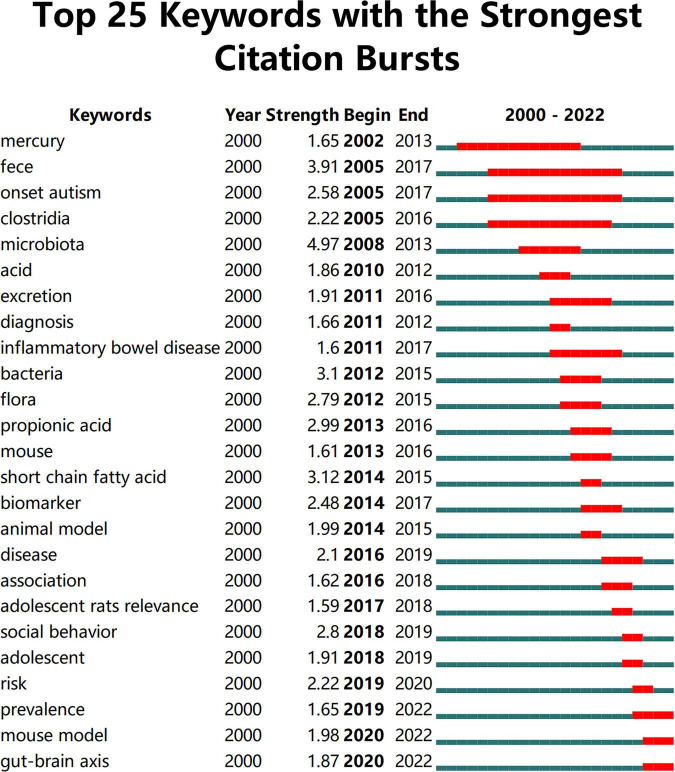
The top 25 keywords with the strongest citation bursts. The strength values indicate the frequency of citation. The red bars indicate the time period in which the keyword appeared.

Keywords often reflect the research hotspots in the field, especially the high-frequency ones. The results include keywords, keyword clusters and burst detection. CiteSpace obtains the keyword network by extracting and analyzing the keywords of articles. After combining synonyms, a total of 373 keywords were obtained, as shown in Figure 7. The nodes represent keywords and the size of a node is proportional to the frequency of keyword occurrence. Except for ASD and gut microbiota, the words children, brain, behavior, propionic acid, chain fatty acid, and feces have high frequencies. Cluster analysis of keywords resulted in 14 clusters (Figure 8), with a modularity Q value of 0.7852 and silhouette value of 0.9175. The modularity Q value is an evaluation index of network modularity, ranging from 0 to 1. When the modularity Q value is higher than 0.3, it means that the network community structure is significant. The silhouette value is a parameter proposed by Kaufman and Rousseeuw in 1990 to evaluate the clustering effect by measuring homogeneity. A silhouette value higher than 0.7 suggests that the clustering result has high reliability. Based on the scores of the modularity Q value and silhouette, this clustering result is reliable. The burst detection results are shown in Figure 9, indicating the research frontiers of this field at a specific time. The strength values indicate the frequency of citation and the red bars indicate the time period in which the keyword appeared. Words of microbiota, feces, short-chain fatty acids, bacteria and propionic acid have high strength.

### Analysis of references

**FIGURE 10:**
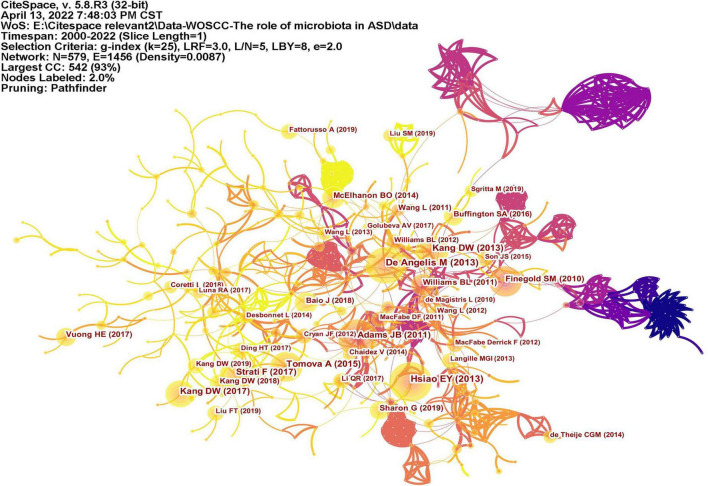
The network of co-cited references. Lines represent articles by two authors were jointly cited by an article. The darker the color, the more times the author’s articles are cited.

**FIGURE 11:**
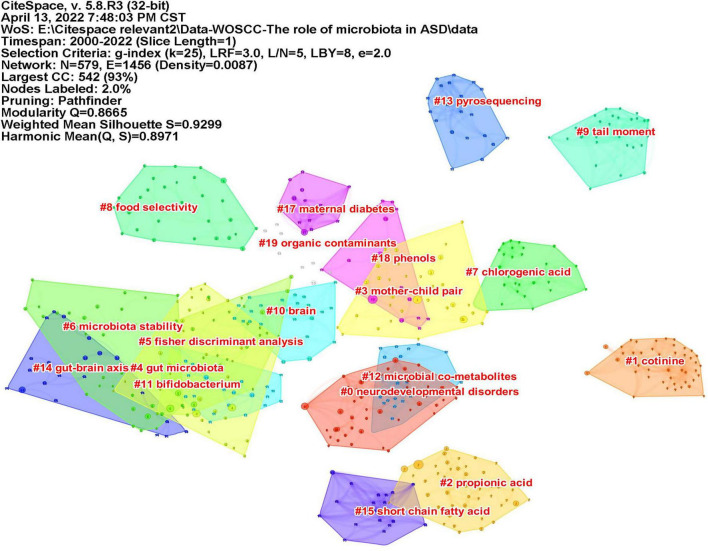
The map of co-cited reference clusters. The clusters are arranged in the descending order of their size.

Co-cited reference means two articles are simultaneously cited by the third article, and the two articles form a co-citation relationship. It reflects intellectual base of the field. The network of co-cited references analysis of 266 articles is shown in Figure 10, with 579 nodes and 1,456 links. The darker the color, the more the article is co-cited. Table 3 lists the top 10 co-cited references. Nineteen clusters were obtained by co-cited reference clustering (Figure 11), with a modularity *Q* value of 0.8665 and a silhouette value of 0.9229. The clusters are arranged in the descending order of their size. Among these clusters, more important clusters were listed: #0 neurodevelopmental disorders, #2 propionic acid, #4 gut microbiota, #10 brain, #11 Bifidobacterium, #12 microbial metabolites, #15 short-chain fatty acids, and #18 phenols.

**TABLE 3:** The top 10 co-cited references.

Rank	Co-cited reference	Count	Centrality
1	Hsiao et al. (18), DOI 10.1016/j.cell.2013.11.02	81	0.02
2	De Angelis et al. (5), DOI 10.1371/journal.pone.007699	80	0.06
3	Kang et al. (6), DOI 10.1371/journal.pone.006832	68	0.18
4	Tomova et al. (53), DOI 10.1016/j.physbeh.2014.10.03	62	0.08
5	Strati et al. (25), DOI 10.1186/s40168-017-0242-	57	0.06
6	Adams et al. (4), DOI 10.1186/1471-230X-11-2	56	0.37
7	Kang et al. (46), DOI 10.1186/s40168-016-0225-	53	0.01
8	Finegold et al. (8), DOI 10.1016/j.anaerobe.2010.06.00	48	0.15
9	Sharon et al. (17), DOI 10.1016/j.cell.2019.05.00	40	0.01
10	Vuong et al. (10), DOI 10.1016/j.biopsych.2016.08.02	39	0.01

## Discussion

### General information

In terms of the number of publications, research on ASD and microbiota began to increase after 2010 and is still on the rise, which is related to the social attention to ASD and the development of detection technology. It has been more than 100 years since autism was first identified, and it was not until 2008 that the United Nations recognized the first World Autism Awareness Day. In 2011, China and the United States jointly established the world’s largest genome database of autism patients, which will bring new opportunities for autism genomics research. In terms of countries and institutions, the United States takes a dominant place in this field, accounting for 32% of the total number of publications. Among the top 10 institutions, four are from the United States. Although China and Italy have a large number of publications, the research institutions are relatively scattered. For the distribution of authors, Afaf El-Ansary from Saudi Arabia published the most articles with an h-index of 25, followed by JAMES B ADAMS of the United States with an h-index of 44. Although James published nearly half as many articles as Afaf, his h-index is significantly higher than that of Afaf, suggesting that James’ articles have a greater impact on the field. According to Price’s Law (from PRICE**⋅**D), the minimum number of publications of core authors is *N* = 0.749$\sqrt{M\,m\,a\,x}$, and *M max* is the number of publications of the most productive authors. It turns out that N≈3. Among the 366 authors, 27 authors published three or more papers, and they published a total of 127 papers, accounting for 47.7% of the total publications, nearly half. It can be seen that research scholars in this field are relatively concentrated.

### Intellectual base and research frontiers

#### Intellectual base

The intellectual base of this field can be seen from co-cited references. The most co-cited article was published by Hsiao et al. (18), which was the first report of intestinal barrier impairment in the offspring of MIA mice with ASD-like behavior and has not been reported before. After oral administration of the human commensal *Bacteroides fragilis*, the gut permeability was corrected, the composition of the microbiota was altered, and the deficits in communication, stereotypic, anxiety-like and sensorimotor behaviors were all alleviated. It also regulated the serum levels of the metabolite 4EPS. These findings provide new evidence for the gut-brain link in ASD and theoretical support for gut microbiota and neurodevelopmental diseases. This groundbreaking study combines animal models, gut microbiota and metabolomics to mechanistically understand the impact of gut microbiota on disease. The research presents a transformative concept, that is, gut microbiota is associated with ASD, and it affects the immune, metabolic and nervous systems. Furthermore, these findings suggest that microbial therapy may be a promising treatment for ASD.

The article with the highest centrality is Adams et al. (4), which explores the relationship between GI symptoms, microbiota and disease severity in children with ASD. This finding indicated that gastrointestinal symptoms in children with ASD were related to the severity of the disease, and changes in the composition of the gut microbiota and metabolome may contribute to both GI and CNS symptoms.

The topics of intellectual base in this field can be seen from the clustering results of co-cited references. ASD is a neurodevelopmental disorder (#0 neurodevelopmental disorder, #10 brain), and a number of studies have described GI symptoms in ASD. Research on the gut microbiota (#4 gut microbiota) and ASD is based on the “gut-brain axis” theory (#14 gut-brain chain), which refers to the two-way pathway formed between the central nervous system (CNS) and the enteric nervous system (ENS), involving nerves, endocrinology, and immunity. Both clinical trials and preclinical research reported that the composition of gut microbiota in ASD was abnormal; for example, the level of probiotic Bifidobacterium (#11 bifidobacterium) was significantly reduced in ASD (5). Metabolites of microbiota (#12 microbial metabolites) are also closely related to ASD. SCFAs (#15 short-chain fatty acid) produced by microorganisms, mainly consisting of acetic acid (AA), butyric acid (BTA), and propionic acid (PPA), can enter the circulatory system and send signals to the brain. Propionic acid (#2 propionic acid) is thought to be associated with ASD. When PPA was injected into the brains of mice, the mice exhibited autism-like behaviors (19). *P*-Cresol is a phenolic compound (#18 phenols). Increased levels of *p*-cresol were observed in the urine and feces of ASD patients (20, 21), and the urinary levels were correlated with their clinical profile severity (22). Mice exposed to *p*-cresol exhibit persistent ASD core symptoms, and this effect depends on changes in the gut microbiota (23). Therefore, the research in this field is based on the theory of the gut-brain axis, and alterations in gut microbiota and its metabolites affect the nervous system and, thus, affect behaviors.

### Research frontiers

#### Composition of gut microbiota

Both clinical trials and preclinical studies have shown significant changes in gut microbiota composition in ASD children and animals. In terms of phylum, the amount and ratio of *Firmicutes* and *Bacteroidetes* and the amount of *Proteobacteria* in ASD children were abnormal compared with those in healthy children (24–27). Genus and species levels of gut microbiota in ASD were also abnormal, including *Bifidobacterium*, *Lactobacillus*, *Bacteroides*, *Clostridium*, *Vibrio desulfurization*, *Akkermansia*, *Roseburia*, *Atopobium*, *Enterobacter*, *Dorea*, *Sutterella*, *Ruminococcus* and so on (28, 29). Among them, the genus *Clostridium* is the focus of research. The abundance of *Clostridium* increased in ASD children (30–33), and after treatment with vancomycin, the levels of *Clostridium* decreased, and ASD symptoms improved (34). In addition, the *Clostridium* family can synthesize ASD-related toxic compounds, such as phenols, *p*-cresol and certain indole derivatives (35), and produce short-chain fatty acids, such as propionic acid (36). Regarding the relationship between *Clostridium* and ASD, scientists have studied relevant treatments. Abuaish et al. (37) found that fecal microbiota transplantation or *Bifidobacterium* treatment could restore the level of *Clostridium spp.* and normalize the expression of brain-derived neurotrophic factor (BDNF) in the hippocampus of PPA-treated mice. Juntao Cai and his team are working on a vaccine against *Clostridium bolteae* (38).

#### Metabolites

The gut microbiota can produce a large number of metabolites, including SCFAs, bile acids, choline metabolites, phenols, benzoyl and phenyl derivatives, indole derivatives, vitamins, lipids, and polyamines. Studies on the microbiota of autistic children and rodent models have found abnormal levels of metabolites. SCFAs are considered key mediators in most research. Research revealed that SCFAs may be directly or indirectly involved in communication along the microbiota-gut-brain axis due to their neural activity properties and their influences on other gut-brain signaling pathways, including the immune and endocrine systems (39, 40). In preclinical studies, SCFAs were considered to have an impact on gastrointestinal motility (41). It may be mediated through activation of SCFA receptors (42), release of enterohormonal peptide YY (41) or release of serotonin from enterochromaffin cells induced by SCFAs (43). Among SCFAs, PPA is the most studied. Using valproate during pregnancy significantly increases the risk of ASD in offspring (44). Intraventricular injection of PPA in rats induced abnormal motor movements, repetitive interests, electrographic changes, cognitive deficits, perseveration, and impaired social interactions. The brain tissues showed many neurochemical changes associated with ASD, including congenital neuroinflammation, increased oxidative stress, glutathione depletion, and altered phospholipid/acylcarnitine profiles (36).

#### Therapy

Microbiota transfer therapy (MTT) is regarded as a potential treatment to modulate disturbances in the gut microbiota and alleviate the behavioral and GI symptoms of ASD. Fecal microbiota transplantation (FMT) is a critical step. Abuaish et al. (37, 45) demonstrated that FMT treatment could improve the social behavior of PPA rats, restore the balance of *Clostridium faecalis*, and affect neural biochemistry. In a clinical trial, Dae-Wook Kang’s team treated 18 ASD children for 9–10 weeks with MTT therapy (FMT for 7–8 weeks). At the end of treatment, behavioral deficits and GI symptoms were ameliorated, and the diversity of gut microbiota and the abundance of beneficial bacteria increased. These changes were maintained 8 weeks after the end of the trial (46), and most of them were maintained after 2 years (47), indicating the long-term efficacy of MTT. Another clinical study by his team found significant changes in blood metabolites in children with ASD after MTT treatment, but not in fecal detection (48). To date, the efficacy of microbiota therapy on ASD has indeed achieved some encouraging results, but not all of them have a positive effect on ASD (49). There are still some defects, such as the unidentified optimal species of bacteria and its dose, limited sample size of clinical studies, and no large RCT studies or placebo group setting. The final outcome of ASD with FMT intervention has not yet been concluded.

### Others

The prevalence and mouse models in the results, including keyword citation burst, may be related to the lag of epidemiological studies on ASD and the lack of ideal animal models. Among the 144 epidemiological studies of ASD reviewed by Eric Fombonne (50), half were published since 2012, and 22 of the 37 countries included findings published in the last 5 years. Animal experiments are an important part of preclinical research, and the establishment of a reliable animal model is important for the study of pathology and exploration of possible treatments. Currently, the most commonly used ASD animal models include MIA models, VPA models, and BTBRT+Itpr3tf/J (BTBR) inbred mouse models. However, these animal models can only simulate some characteristics of ASD. In 2016, Zilong Qiu’s team (51) produced MECP2 (a gene strongly related to autism) transgenic monkeys. These transgenic monkeys exhibited ASD-like behaviors, such as social deficits, stereotyped behaviors, anxiety, and relatively weak cognitive abilities. Notably, they exhibited transgenic germline stability. This is the first study to establish a nonhuman primate model with a human autism gene, which indicates that using genetically engineered nonhuman primates to study brain disorders is feasible. However, whether this can be an ideal model for ASD needs further research.

Martínez et al. (52) also published an article on the bibliometric analysis of ASD and gut microbiota. Similarly, the number of publications, authors, institutions and countries of ASD and GM were analyzed from the past to the present. The effect of gut microbiota on ASD behavior and ASD animal models were both mentioned. The main differences between the two articles are as follows. First, the time periods were different. The time range of literature retrieval in this study was from January 1, 1980 to April 1, 2022, while Martínezs’ was from 1992 to 2020. Second, only original articles were included in this study, but Martínezs included all other types, such as review articles, editorial material, meeting abstracts and so on. Third and most importantly, the study mainly focused on the analysis of ASD and GM research results, including the intellectual base, hot spots and frontiers, while Martínezs focused more on the analysis of the literature, such as article types, journals, institutions and authors.

## Conclusion

The study of the link between ASD and gut microbiota is based on the gut-brain axis. The effects of alterations in the composition and metabolites of gut microbiota on ASD behaviors and GI symptoms are the focus. Alterations in gut microbiota composition affect ASD, especially *Clostridium.* Changes in gut microbiota metabolites, especially propionic acid and phenols, also affect ASD. The role of gut microbiota in the pathogenesis of ASD involves neural, immune and metabolic pathways. FMT therapy may be a potential treatment. However, the exact microbial composition and metabolites associated with ASD have not yet been determined, and FMT is still in its infancy. Therefore, this field still has great research potential.

## Data availability statement

The original contributions presented in this study are included in the article/Supplementary material, further inquiries can be directed to the corresponding author.

## Author contributions

XL and RZ conceived and designed the experiments, performed the experiments, analyzed the data, reviewed the drafts of the manuscript, and approved the final draft. XL wrote the original draft. DL performed the experiments, analyzed the data, prepared figures and tables, and approved the final draft. LX and LZ performed the experiments, prepared figures and tables, and approved the final draft. XC conceived the experiment, reviewed and edited the final draft. All authors contributed to the article and approved the submitted version.
